# Supplementary material for: Mechanism of life-long maintenance of neuron identity despite molecular fluctuations
Source: eLife. 2021 Dec 15;10:e66955. doi: 10.7554/eLife.66955 (PMC8735970; doi:10.7554/eLife.66955)
Supplement: Supplementary file 4. [file elife-66955-supp5.docx]

| **Supplementary Table.** ssODNs and guides used in this research. | | |
| --- | --- | --- |
| **ssODN** | **Allele** | **Sequence** |
| 3414 | *gj2089[che-1::GFP::AID]* | TGCTATACGAAGTTATTTTAAACTTACCTTTCCCTTCACGAACGCCGCCGCCTCCGGGCCACCGCTTGATTTTTGGCAGGAAACCATCACGTTCTTCCGGTATGATCGCACCGGTGGCCATCCCACAACTTGTGCCTTGGCCGGAGGTTTGGCTGGATCTTTAGGCATGGATTGAAAGTACAGATTCTCCTTGTAGAGCT |
| 3416 | *gj2062[(ASE_gcy-22_ + flanks)p::che-1::GFP::AID]* | TGTTCTCTAAAATTGAAAAATAAGGAATCAGAATGTTTTTATATATTTTCAGTTATATCACATTTTACGAGCATTTATGTGCCCCACTTCGAATTTGAAGGGCTTCCCATAGACGCGTGATTTTATTTCGAATTTCGAGATTTCGAGAAAATTAAACCGTTTTTTTTCTCTTCTTCTTCAGCTCATCATCGAATCTCACC |
| 3417 | *gj2063[(ASE_gcy-22_)p::che-1::GFP::AID]* | AAGGAATCAGAATGTGTAGGAAGTTGTTAGTAAGTGAAGCCCTTCAATTCATAGATAAGAAGAAACTACGGTATTCGGATTA |
| 3420 | *gj2088[(ΔHD)p::che-1::GFP::AID* | AGCTGAAGAAGAAGAGAAATTGGGAGAGAAGAGATGAATACCGTAGTTTCTTCTTATCTATGAAAATTGTGGCT |
| 3415 | *gj2065[(ASE_che-1_ + flanks)p::gcy-22]* | ATTTTCCTGGTTATCTGACGTTAATTCAGTGATTCAATTGTTCTCTAAAATTGAAAAATAAGGAATCAGAATGTGTAGGAAGTTGTTAGTAAGTGAAGCCACAATTTTCATAGATAAGAAGAAACTACGGTATTCGGATTAAAATCTCTTCTCTCCCAATTTCTCCCTACGTCTTTGAATATTATAGGATTTCACAAAAT |
| 3418 | *gj2064[(ASE_che-1_)p::gcy-22]* | ATCTCGAAATTCGAAATAAAATCACGCGTCTATGGGAAGCCACAATTATTCGAAGTGGGGCACATAAATGCTCGTAAAATGT |
|  |  |  |
| 3579 | *ceh-36(gj2127)* | ATTTGCGGATCCGTCGAGCACTTCGCCTGCCCCCGtttaaaaTAAGTTTTGAATATTCCCTTTTGATTTTTAATTCA |
| 2679 | *gj1959[osm-3::GFP]* | CTTGCTCACCATGACCGGTACCTTGGGATTCAGAGAAGCTAG |
| 2643 | *gj1959[osm-3::GFP]* | TTGTAAAACGACGGCCAGTGAATGGAAGTGCTAGCCTAGG |
| 2660 | *gj1959[osm-3::GFP]* | CGAGCTGTACAAGTAAGAATTCTTCGTGTGTACATTGTGATG |
| 2646 | *gj1959[osm-3::GFP]* | GACCATGATTACGCCAAGCTCATATGGAGGCGGTTGGTCTTATTAC |
|  |  |  |
| **guide** | **Allele** | **Sequence** |
| g2 | *gj2089[che-1::GFP::AID]* | TCTGTACTTTCAATCCGGAA |
| g51 | *gj2062[(ASE_gcy-22_ + flanks)p::che-1::GFP::AID]* | GCTGAAGAAGAAGAGAAATT |
| g52 | *gj2062[(ASE_gcy-22_ + flanks)p::che-1::GFP::AID]* | AATAAGGAATCAGAATGTGT |
| g53 | *gj2063[(ASE_gcy-22_)p::che-1::GFP::AID]* | CTTCTTATCTATGAAAATTG |
| g54 | *gj2088[(ΔHD)p::che-1::GFP::AID]* | TAAGAAGAAACTACGGTATT |
| g55 | *gj2065[(ASE_che-1_+flanks)p::gcy-22]* | ATATTCAAAGACGTAGGTGT |
| g56 | *gj2065[(ASE_che-1_+flanks)p::gcy-22]* | CGTTAATTCAGTGATTCATA |
| g57 | *gj2064[(ASE_che-1_)p::gcy-22]* | TGCCCCACTTCGAATTTGAA |
| g63 | *ceh-36(gj2127)* | GGAATATTCAAAACTTAAAG |
| g66 | *ceh-36(gj2127)* | GGTGGTCATTGTGCATGCGG |
| g50 | *gj1959[osm-3::GFP]* | GAATTATTTGGGATTCAGAG |
